# COVID-19 mRNA vaccine induced rhabdomyolysis and fasciitis

**DOI:** 10.1007/s00415-021-10768-3

**Published:** 2021-08-25

**Authors:** Simon Faissner, Daniel Richter, Ulas Ceylan, Christiane Schneider-Gold, Ralf Gold

**Affiliations:** Department of Neurology, Ruhr-University Bochum, St. Josef-Hospital, Gudrunstr. 56, 44791 Bochum, Germany

Dear Sirs,

Since the end of 2019, the coronavirus disease 2019 (COVID-19) pandemic induced by an infection with severe acute respiratory coronavirus 2 (SARS-CoV-2) has led to millions of deaths worldwide. The unprecedented fast development of a vaccination against SARS-CoV-2 led to the approval of several vaccinations by the authorities since December 2020. Rare severe side effects are sometimes not observed during the pivotal trials but get noticed during daily clinical practice such as thrombotic thrombocytopenia after ChAdOx1 nCov-19 vaccination [1]. Thus, the description of rare side effects is urgently needed to optimize the worldwide COVID-19 vaccination campaign. Here, we report for the first time a case of myopathy with severe rhabdomyolysis and fasciitis following mRNA vaccination against SARS-CoV-2. The patient was treated in the University Hospital of Ruhr-University Bochum and gave written informed consent for the publication of her case.

A 28-year-old healthy female received the first dosage of mRNA vaccination against SARS-CoV-2 (Moderna). Five days later, she complained about muscle pain of her thigh muscles, radiating to the lower legs, accompanied by an asymmetrical weakness of the lower limbs. Seven days following vaccination, a blood test revealed marked elevation of creatine kinase and transaminases. At first presentation, she had a mild predominantly left-sided weakness of hip flexor and knee extension (MRC 4-/5 vs. MRC 4/5) with marked subcutaneous leg edema (Fig. 1a), the creatine kinase was 17,959 U/l (normal range 26–140 U/l). There had been no preceding muscular symptoms such as exercise intolerance or anesthetic reactions that might have suggested a preceding predisposition to develop rhabdomyolysis. There was also no relevant family history. Immediate workup excluded infection with hepatitis viruses, EBV or CMV. An echocardiography and a thoracic computed tomography were unremarkable. The patient was immediately treated with high volume normal saline infusion and urine alkalization. Renal function gradually worsened with a creatinine of 1.02 mg/dl (75 ml/min glomerular filtration rate according to Cockcroft–Gault) on day 5 of in-hospital treatment. Moreover, hypocalcemia of 1.97 mmol/l and moderate hypophosphatemia of 0.38 mmol/l were detected at first presentation, which gradually resolved within 4 days; parathormone was normal. Antibodies associated with myositis or myopathy were all negative (Mi-2a/b, TIF1g, MDA5, NFP2, SAE1, Ku, PM-Scl100, PM-Scl75, Jo-1, SRP, PL-7, PL-12, EJ, OJ, Ro-52, cN-1A). An MRI of the thigh muscles, performed on the following day, showed left-dominant edematous signal alterations with contrast enhancement of the quadriceps muscles sparing the M. rectus femoris, and diffuse subcutaneous fluid retention with contrast enhancement, suggestive of fasciitis (Fig. 1b, c). Electromyography of the left rectus muscle showed positive sharp waves and fibrillations with small and partly polyphasic motor unit potentials, compatible with an acute myopathy or myositis. The patient was treated with an i.v. cycle of 250 mg methylprednisolone over 2 days, leading to complete remission of paresis, leg pain and remission of leg edema within days, followed by oral tapering (60 mg methylprednisolone orally, tapered over 6 days). Four weeks after onset, creatine kinase was normal, the weakness and leg edema were gone, and she could do jogging again.

**Fig. 1:**
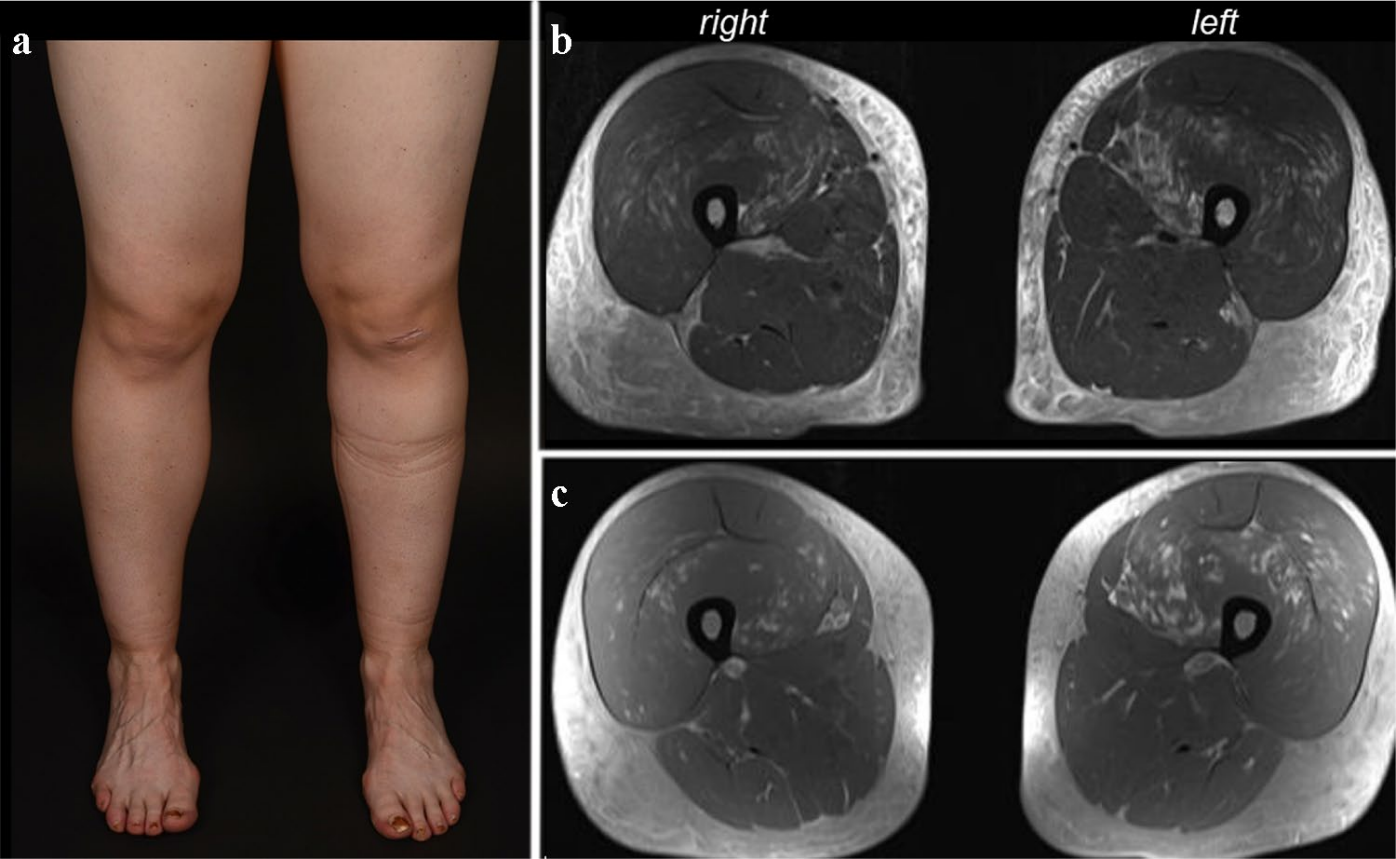
COVID-19 mRNA vaccine induced myopathy with rhabdomyolysis and fasciitis in a 28-year-old female. **a** Edematous swelling of both legs. **b** Transversal MRI of the thigh muscles in T2 and **c** T1 post-contrast shows left dominant signal alterations of the quadriceps muscles with contrast enhancement and subcutaneous edema suggestive of accompanying fasciitis

COVID-19 mRNA vaccine-associated side effects include pain, redness and pain at the injection site, fatigue, headache, myalgia or arthralgia [2, 3]. One case described local COVID-19 vaccine-related myopathy in the deltoid muscle with probable myositis [4]. Recently, another case report described severe rhabdomyolysis 1 day after BioNTech/Pfizer COVID-19 vaccine administration with creatinine kinase levels of up to 22,000 U/l [5]; however, without signs of myopathy or fasciitis as found in the patient presented here. COVID-19 can induce a broad range of neurological symptoms including encephalitis, encephalopathy, cranial neuropathy, Guillain–Barré syndrome, and myositis/rhabdomyolysis. As of September 2020, Paliwal et al. found nine cases of myositis/rhabdomyolysis induced by natural COVID-19 disease [6]. One report documented a patient with myopericarditis and myositis with similar subcutaneous edema and signal alterations of the thigh muscles suggestive of myositis with creatine kinase elevations [7] as observed in our patient following vaccination, suggesting similar pathomechanisms.

In summary, we present a new and so far unknown complication of mRNA vaccination against SARS-CoV-2. Clinicians should be vigilant especially in patients developing myalgia with paresis following COVID-19 vaccination to detect rhabdomyolysis and start treatment without delay.

## Data Availability

Data are available from the corresponding author upon reasonable request.
